# Multisector collaborations at children’s hospitals to address social drivers of health

**DOI:** 10.1093/intqhc/mzaf067

**Published:** 2025-07-18

**Authors:** Ulfat Shaikh, Melissa Gosdin, Elizabeth Helmke

**Affiliations:** Pediatrics, University of California Davis School of Medicine, 2516 Stockton Blvd, Suite 340, Sacramento, CA 95817, United States; Center for Healthcare Policy and Research, University of California Davis, 4900 Broadway, Suite 1430, Sacramento, CA 95820, United States; University of California Davis School of Medicine, 4610 X St, Sacramento, CA 95817, United States

**Keywords:** hospitals, pediatric, child health services, intersectoral collaboration, pediatrics, child health, community health services, community networks, social determinants of health, healthcare disparities

## Abstract

**Background:**

Child health is influenced by a range of social drivers. This necessitates a multipronged approach to pediatric care with collaborative efforts of multiple sectors. Children’s hospitals have unique expertise and resources to identify pressing issues in child health and partner with community organizations and local governing bodies to address gaps in child health. The goal of this study was to identify multisector collaborations that children’s hospitals in the USA engage in, facilitators and challenges of these efforts, and best practices that hospitals can employ to implement and sustain such collaborations.

**Methods:**

An environmental scan was conducted utilizing the following approaches: a content analysis of Community Health Needs Assessments (CHNAs) and implementation strategies at 35 children’s hospitals, and semistructured qualitative interviews with leaders who manage community partnerships at 14 select children’s hospitals. This purposive sample was selected for national representation, including geographic location, size, and type of pediatric hospital. Qualitative relational analysis enabled exploration of descriptive and interpretive meanings within CHNA documents. Audio recordings were transcribed, the interview guide focused on domains of the PRISM (practical robust implementation and sustainability model) implementation science framework, and interviews were conducted and analyzed by a team of qualitative researchers.

**Results:**

Most hospitals engaged in multisector collaborations that addressed health disparities. The most frequently identified priorities included mental and behavioral health, access to health services, neighborhood safety and violence prevention, early childhood education, and chronic disease prevention. Key challenges were limited funding for multisector collaboration, shortage of staff with training and experience in multisector work, and variable readiness of community partners. Facilitators included adequate staffing and funding, community trust, and building on existing partnerships. All hospitals highlighted the crucial need to build trust within the community as a key factor to implement successful multisector collaborations. Best practices included examining the hospital’s internal organization to avoid duplication of efforts, leveraging existing hospital resources to support local initiatives, incorporating community partners and financially supporting their efforts, creating avenues for bidirectional communication with community partners, measuring and tracking effectiveness of collaborations, and developing infrastructures to keep projects moving forward despite staff turnover.

**Conclusion:**

Efforts to transform child health care from sick-care systems to community-integrated systems require children’s hospitals to partner with community-based organizations to extend their reach and effectiveness. These findings can be used to develop strategies to implement and sustain future multisector collaborations at children’s hospitals to effectively leverage their existing strengths and resources.

## Introduction

Clinical care accounts for only 20% of health outcomes while social drivers of health, health behaviors, and the physical environment contribute to 80% of health outcomes [1]. Child health is influenced by a range of factors such as nutrition, education, housing, household finances, caregiver employment, neighborhood safety, transportation, language, health literacy, and health care services. This necessitates a multipronged approach to pediatric care, the effectiveness of which is multiplied through the collaborative efforts of multiple sectors [2]. Children’s hospitals have unique expertise and resources to identify pressing issues in child health and partner with community organizations and local governing bodies to address persistent gaps in child health. Such multisector collaborations can play a key role in health systems transformation, especially in value-based financing models where hospital reimbursements are based on quality measures and health outcomes [3].

Potential partners for multisector collaborations to enhance child health include local school districts, social services (e.g. child protective services, crisis nurseries, foster care services, Women, Infant and Children Programs, food banks), justice (e.g. law enforcement, legal aid organizations, juvenile justice systems), housing, transportation, faith-based organizations, local or county health and public health departments, parks and recreation departments, refugee and immigrant services, chambers of commerce, and local businesses.

Assessing factors that facilitate the implementation of multisector collaborations could help children’s hospitals develop effective strategies to adopt, scale up, and sustain such efforts. Since 2010, the Affordable Care Act has required hospitals with nonprofit tax-exempt status in the USA to conduct a Community Health Needs Assessments (CHNAs) at least once every 3 years [4]. The purpose is to identify community needs and develop an implementation strategy to address high priority needs. The findings of the CHNA and the implementation strategy are documented in a report and made available on the hospital’s website. The overall goal of this study was to utilize CHNAs, CHNA implementation strategies, and in-depth interviews to identify factors that play a role in the readiness of children’s hospitals in the USA to engage in multisector collaborations within their communities. The objectives of this study were to determine how organizational structures of children’s hospitals enhance their engagement in multisector collaborations and identify key facilitators and challenges that influence such collaborations.

## Methods

Multisector collaborations for the purpose of this study were defined as partnerships between a children’s hospital and one or more organizations whose primary purpose does not involve health care delivery. The PRISM (practical robust implementation and sustainability model) implementation science framework was utilized to conduct an environmental scan that addressed the following research questions: Among a sample of children’s hospitals in the USA that are engaged in multisector collaborations: (i) What types of multisector collaborations are hospitals involved in? (ii) What is their organizational structure for leading, conducting and evaluating multisector collaborations? (iii) What are the outcomes, facilitators, and challenges of these multisector collaborations? [5].

A two-pronged qualitative research approach was employed to gain these insights: (i) a content analysis of CHNAs and CHNA implementation strategies at 35 children’s hospitals and (ii) semistructured qualitative interviews with leaders who manage community partnerships at 14 select hospitals. Based on feedback from four hospital leaders not included in this sample, the coding and interview guides were adjusted to better reflect the scope of multisector collaborations and to gain a deeper understanding of hospitals’ experiences.

For the content analysis of CHNAs and implementation strategies, a purposive sample of 35 hospitals were included from a total of 218 children’s hospitals in the USA that are members of the Children’s Hospital Association. This sample was intentionally selected for diverse national representation, including geographic location, size, and type of pediatric hospital. Purposive sampling involves intentionally selecting participants based on characteristics that will yield the most information [6].

Qualitative relational analysis enabled the exploration of both manifest and latent meanings (descriptive and interpretive) within these documents [7]. The types of codes used were inductive (new and emergent codes) and deductive (*a priori* codes based on the literature and existing theoretical frameworks). For the coding process, the unit of analysis was first identified, then open coding was used (initial read through of one CHNA and implementation strategy), followed by each coder creating an initial list of codes to decontextualize the data into meaning units. Coders compared codes, with one author serving as a tiebreaker. This process was then repeated. After an initial list of codes and definitions was agreed upon, a codebook (coding protocol) was created, and the coders began the process of coding in Dedoose version 9.0 [8]. Coders met periodically to review changes to the codebook and to discuss and compare coded CHNA and implementation strategies.

To facilitate semistructured in-depth interviews, a set of research questions was developed based on guidance from four hospital leaders not included in this sample, aimed at understanding factors influencing readiness for multisector collaborations. These questions delved into the impact of organizational structure on collaborations to identify key facilitators, challenges, and best practices in multisector collaborations. A purposive sample of leaders at 14 Children’s Hospital Association member hospitals was selected for individual 60-min in-depth online video interviews. Verbal informed consent was obtained from participants prior to beginning the interview and documented by the interviewer as per human subjects’ research ethics approval requirements.

Interviews were conducted and analyzed by a qualitative researcher, and audio recordings were transcribed verbatim by a professional transcription service. The interview guide consisted of questions focused on the five domains of the RE-AIM planning and evaluation framework: reach, effectiveness, adoption, maintenance and implementation [9]. Questions included the types of multisector collaborations that hospitals are involved in, focus areas being addressed, collaborators, trajectory of activities, prioritization of initiatives, challenges, needs and gaps, workflow, communication, information sharing, resources and support, patient and family engagement, measures and metrics, promising interventions, and opportunities. Interviews were conducted until data saturation occurred and no new concepts were identified [10].

## Results

The 35 children’s hospitals included in the content analysis spanned a range of characteristics, described in Table 1. All hospitals were engaged in some level of multisector collaboration. Efforts to reduce health disparities emerged as a primary focal point of these collaborations.

**Table 1.** Key characteristics of children’s hospitals included in content analysis of Community Health Needs Assessments and implementation strategies (*N* = 35)

Region	% (*n*)
Midwest	34.3 (12)
Northeast	14.3 (5)
South	25.7 (9)
West	25.7 (9)
**Annual admissions**	
<5000	17.1 (6)
5000–9999	37.2 (13)
10 000–14,999	28.6 (10)
15 000+	17.1 (6)
**Number of beds**	
<150	20 (7)
150–299	34.3 (12)
300–449	34.3 (12)
450+	11.4 (4)
**Hospital type**	
Acute-care freestanding children’s hospital	66 (23)
Children’s hospital within hospital/system	34 (12)

### Priority areas for multisector collaborations

Priority areas for multisector collaborations at most hospitals included in this study were determined by their CHNAs. The most frequently identified priorities included mental and behavioral health, access to health services, neighborhood safety and violence prevention, early childhood education, and chronic disease prevention (specifically, obesity, diabetes, and asthma). Other issues identified were incarceration, climate change, and parental education. Racial inequities as a source of health disparities were mentioned in every CHNA analyzed in this study. Some hospitals addressed it as a standalone priority area, while others integrated health disparities into new or existing strategies.

There was variation in the number of priority areas addressed by hospitals, with some hospitals selecting 1–2 focus areas and others addressing up to nine areas. Selection of priority areas was determined by organizational capacity, ongoing investments, alignment with their mission, magnitude of the problem, local disparities, existing interventions, and likelihood of sustainability. Several CHNA implementation strategies included place-based interventions to address needs within a particular zip code or community in the hospital’s service area. Needs identified in CHNAs aligned with implementation strategies at 94% (33/35) of children’s hospitals.

### Facilitators to multisector collaborations

**Figure 1:**
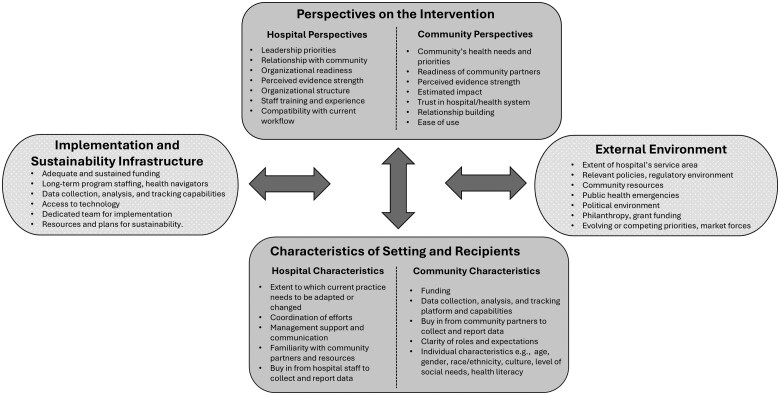
Utilizing the PRISM to identify factors affecting implementation of multisector collaborations at children’s hospitals

Children’s hospitals included in the analyses developed partnerships with a range of community entities, including local school districts, parks and recreation departments, food banks, legal aid organizations, and churches (Supplementary Table S1). These partnerships resulted in changes in policies and improvements in measurable outcomes. While interviewees universally recognized staffing and funding as key facilitators, they cited a variety of other factors based on their hospital’s organizational structure or unique community context as noted in Fig. 1. For instance, some hospitals benefitted from a tight-knit community with well-integrated community organizations. Other hospitals were geographically located close to their community partners, allowing for convenient programming, while some housed their partners on their campus.

Hospitals varied significantly in the size of their community engagement departments, philanthropic and grant support, research and data analysis assistance, and leadership buy-in for community engagement work. Additional facilitators of multisector collaborations are listed in Table 2 and included community trust, existing strong partnerships to build on, hiring community engagement specialists to prepare the hospital to work in the community, quality improvement training, conducting design sessions with community members, and training community collaborators in collecting data to measure and track outcomes.

**Table 2.** Facilitators to multisector collaborations at children’s hospitals

Summary of themes related to facilitators	Sample of illustrative quotes
*Reach* Investment of time and effort to build community trust Design sessions with community members	“Community trust is entering into meetings and conversations, even if you’re the unknown entity by saying, ‘This is what we think is happening based on the data that we see. Is this true? If so, why? Or how? Or if not, tell us about that.’” “We realized very, very quickly that this isn’t a matter of building trust. It’s a far more daunting challenge of overcoming mistrust.” “Let’s bring in childcare providers … But let’s make sure to host that listening session to makes this transit-friendly and parking friendly.” “Two elements to our strategy: person-centered design, community-centered design. And as part of that, we run design sessions where we will hire a nonprofit from the community to run formal design sessions with community members.”
*Effectiveness* Effort to reduce disparities in social determinants of health Training community collaborators in data collection and reporting for outcome measurement	“… the health department, in particular is a wonderful partner. They are very attuned to social determinants of health. So, any time they pull together multisector collaborators, they’re leading with that in mind.” “We’ve worked with programs in trying to develop common metrics across all of the programs … One thing that we have attempted to do is to encourage all programs … to consider the extent to which they are strengthening families … We’ve employed the Center for the Study of Social Policy’s Strengthening Families’ Protective Factors framework.”
*Adoption* Existing strong partnerships Range of community partnerships, including school districts, parks and recreation, food banks, legal aid organizations, churches Institutional support and leadership buy-in	“We’ve had longstanding relationship with [county] Health Department, and they are our WIC partner. We’ve had longstanding relationships with public schools. We have longstanding partnerships with Safe Kids Worldwide … It takes a lot of commitment on both parties to really come to work together.” “We’re very, very lucky that we have really supportive leadership. I think because we are successful with philanthropy, this doesn’t affect the hospital’s overall budget … Once we show our senior leadership some of the outcomes … it’s very well received. Also, we work closely with our marketing communications to share these successes.”
*Implementation* Community engagement specialists Engaging experienced staff to lead initiatives Adequate staffing and funding Streamlined points of contact Quality improvement training Generosity towards community partners	“I was able to hire a very experienced and well-known individual in our community that had already had trust built up with many of our community organizations.” “Each program has a face to it. So, our public schools knows that we’re doing food work, they know I'm the face of that, and so they have that identification there.” “I give a shoutout to our Foundation on this one. They know potential funders and donors, and that’s where our brand recognition does really work in our favor, when they know who to approach to talk to about philanthropic funding.” “What’s been really helpful most recently is that our quality improvement department has offered training in organizational and operational excellence.” “We’ve taken all our quality improvement courses that we do internally … we run them externally for quality improvement. So, we’re now about to start our ninth year of community leaders, … [taking] this course.” “How we show up for a community who needs us, means being ready for whatever it is that they ask for in a just-in-time kind of way … It’s an open collaborative agreement … They’ll say, ‘Hey, we’re hosting a health fair for our new move-in family. Could you show up and table?’ Of course. And then vice versa, we’ll say, ‘Hey, we’re doing an event with other community partners, and we’d love to showcase your good work as an example of how to start a not-for-profit.’”
*Maintenance* Utilizing resources and services located close to the hospital Outcomes tracking	“If a need is revealed, [families] can come upstairs to our resource center, which is a suite of offices that we host CBOs on site. It’s the epitome of a warm handoff.” “We collaborate with our neighbors quite a bit … It’s so easy to just to zip across the street when a call happens … Let’s say the housing center says, “Hey, I've just gotten a new family to move into a unit. They’re new to the area. Could you bring over some jackets?” … So, this is how we show up for our neighbors in the community.” “Six years ago, we added a data analytics team, and that has propelled our work, no doubt.” “We use CBISA, which is a community benefits software … we train superusers throughout the organization that may be doing community programs.”

### Challenges to multisector collaborations

The three most frequently reported challenges were limited funding for multisector collaborations, shortage of staff with training and experience in multisector work, and variation in the readiness of community partners. As listed in Table 3, other challenges reported were lack of familiarity with community partners and resources, limited infrastructure to implement and scale up programs, lack of leadership buy-in for community-based work, evolving community priorities, and siloed organizational structure.

**Table 3.** Challenges to multisector collaborations at children’s hospitals

Summary of themes related to challenges	Sample of illustrative quotes
*Reach* Variation in readiness of community partners Time and effort needed to build trust Large service areas served by some hospitals	“I think readiness is a key component to partnerships. People have ideas, but are both organizations in terms of staffing and finances ready to really kick that off?” “In different communities, it may take longer [to build trust] if some of those key players are not known.” “How do I cover a million square miles and engender trust and goodwill when the last three years I've only met you via Zoom?”
*Effectiveness* Challenges with data collection and tracking Need to build long-term community relationships Challenges with retaining long-term program staffing	“A lot of the collaborations we’re involved in are other hospitals that use a different EMR- with much more data capabilities. That’s the biggest challenge is that we don’t have the data, we’re not putting it in, we don’t capture it well, we can’t use the tools that these other sites have been able to use because we’re on a whole different platform.” “We know that it takes time to build relationships and get to a place of deliverables in a relationship or a program … We know that to move the dial and to really show impact, it takes a minimum of seven to 10 years. It’s really hard in our community health world to make that compelling, when we’re short of those measures.”
*Adoption* Lack of institutional support or leadership buy-in Limited funding for multisector collaborations Lack of recognition of community engagement work Evolving or competing community or hospital priorities, including public health emergencies	“I think a lot of the stuff that we do could be considered non-value added. It’s a difficult conversation to have.” “Sometimes partnerships will drift apart because your interests and agendas are different.”
*Implementation* Lack of dedicated hospital resources (staff, programming space, funding) Unclear expectations about hospital and community partners’ role in the collaboration	“It’s the bandwidth and FTE resource to help support and facilitate the work that we do.” “There were many people interested in the community, but it was almost like each organization took a different piece … Trying to figure out seed money for funding and what was each organization’s role was going to be, and how is it different than some of the things that were already in place in the community.”
*Maintenance* Limitations in community resources or infrastructure to address or scale up specific priority areas Shortage of long-term staff with training and experience Siloes in the organizational structures within hospitals	“We think about our shelter system, they don’t have enough staff. Their staff turns over very frequently because they can’t pay a competitive wage … That has been really hard to overcome.” “You need to hire someone who’s going to be willing to be here for more than a hot minute, because our work is relationship-based.” “We do have individual departments and divisions and clinicians who are creating partnerships. I think a constant challenge is how to have wonderful distributed leadership, but then actually not have a thousand points of uncoordinated efforts that have marginal impact.”

Data collection obstacles were highlighted by most hospital leaders interviewed, and included paucity of staff to gather data, lack of a consistent data collection system or platform, and challenges with adherence of hospital staff or community partners to track and report data. Several hospitals identified the impact of the COVID-19 pandemic on multisector collaborations, from expanding opportunities for collaboration (vaccine administration, promoting community resources to families impacted financially), to widening existing health disparities and hampering in-person community partnerships.

### Best practices in the implementation of multisector collaborations by children’s hospitals

All hospitals highlighted the crucial need to build trust within the community as a key factor to implement successful multisector collaborations. To this end, all children’s hospitals in the sample engaged community members in developing the CHNA utilizing survey and focus groups or brainstorming sessions with community partners to identify pressing community health needs. Sixty-six percent (23/35) used community member input in developing implementation strategies through direct leadership in multisector initiatives or the creation of community advisory boards. Other hospitals served community partners by showing up as dependable organizations, consistently supporting local initiatives, donating to community needs, and compensating community partners for their efforts. Taking a collaborative, rather than prescriptive approach to partnerships also enhanced community willingness to work with hospitals.

As listed in Table 4, organizational practices that improved the efficiency of collaborative efforts included examining the hospital’s internal organization to avoid duplication of efforts, creating avenues for bidirectional communication with community partners, leveraging existing hospital resources, and developing infrastructure to keep projects moving forward despite staff turnover. A consistent theme among children’s hospitals that successfully maintained multisector collaborations was that they viewed these efforts as investments in long-term relationships with the surrounding community. Some hospitals prioritized collaborators with whom they shared long and effective partnerships, since these established collaborations could serve as launchpads for future initiatives. These hospitals also highlighted the importance of administrative support for long-term initiatives rather than only prioritizing collaborations that produce quick outcomes.

**Table 4.** Best practices in the implementation of multisector collaborations at children’s hospitals

Summary of themes related to best practices	Sample of illustrative quotes
*Reach* Build trust within the community Elicit community member feedback and input on community health issues and opportunities for collaboration Partner with networks of trusted/well-respected community organizations Emphasize a collaborative rather than prescriptive approach to multisector collaborations	“…Whenever we meet with the leaders in new communities, we go to them with the approach of what are your goals for your community? What are you all working on right now? … how can we be helpful to you” “They [community partners] actually say that’s why they trusted us, because we didn’t come in there to say, “This is what we’d like to do in your community.” We came in there to say, “This is our understanding this is what you are trying to achieve, and here’s how we might be able to help.” “It’s a far more daunting challenge of overcoming mistrust … The first thing that we did was take our budget and open to the public … We were totally transparent with respect to how dollars were being spent. And the second thing that we did was to embed community members in every element of the initiative’s governance and leadership structure. And relatedly, any community member who is working with us, we pay them for their time and effort.” “We say, ‘here’s the burden of disease in 250 indices of pediatric health.’ But then we go to community partners and members, and parents, guardians, and caregivers and say, ‘This is what the data says. Is this true for you?’” “Part of building trust with community is going hand-in-hand with another trusted community partner that has already done the work … they are the entity to help open the door” “It wasn’t just Children’s coming in and say, ‘Hey, this is how we do it. And we want you to do it this way.’ It was, ‘What works for the hospital, what works for the school, and how can we fuse those two things together to work best in each environment?’”
*Effectiveness* Ability to respond and continue multisector work despite public health emergencies Obtain assistance from community partner or research center to collect data Utilize technology, electronic health records, or dashboards to collect key metrics Evaluate the quality of multisector collaborations, conduct social network analysis to measure engagement with the initiative, evaluate broader indicators of community health	“When COVID hit, we were just getting everything well-oiled and then all of a sudden, schools were shutting down. We had to pivot to telehealth, which we did within weeks. So, we still serviced children through the school-based behavioral health program, even though the schools were shut down.” “We are partnering with very strong community-based organizations, …, each of whom gather the type of data that we need in order to show community impact, impact at the population level, and follow trends on a timely basis.” “The vast majority of my programs have dashboards that track a lot of our metrics and outcomes … We not only have the ability to look at data over a period of time and track change over time, but these dashboards allow us to actually intervene in live time” “We tie many of these metrics and some of the outcomes for these to our quality improvement dashboards that also go up to quality executive and to our CEO.” “We’ve used social network analysis as a strategy to look at the extent to which our programs are connected to each other.” “I think it’s really easy to actually make the connections to the quality pillar, because we know that by addressing social needs, it will influence health outcomes even more than maybe what happens in the clinic.”
*Adoption* Alignment of multisector collaborations with hospitals’ strategic plan Establish shared goals Set expectations of what hospital and community partner can contribute, and create timelines Streamlined points of contact Messaging that is sensitive to community’s values and beliefs Provide credit to community partners	“This last strategic plan that we did in 2020 had an entire pillar dedicated to community impact.” “Working with schools, they’re all different. They all have school boards. They all have superintendents and principals … As long as you stay focused on the true north and those shared interests …” “I think the best partnerships that we have are the ones where we have really spelled out expectations and roles.” “We do enter into MOUs with all partnering agencies. So, we go through a contract where it outlines roles, responsibilities, expectations, what access to data.” “It helps when, if you’re trying to do multiple initiatives with the school system, you funnel that through one person.” “These are the things that we do really well … Here are the things that other teams do at [hospital], because they do it better than us. And here is where we paid or gifted to this community-based organization because they know how to do it even better than we do.”
*Implementation* Avoid duplicating existing services in the community Developing community engagement departments or offices at children’s hospitals Creating avenues for bi-directional communication with community partners Providing financial support to partners and communities Funding for community engagement activities through grants and philanthropy, grant specialists that write and manage grants and conduct program evaluations Advocacy for government funding for specific initiatives Use quality improvement methods such as Plan-Do-Study-Act cycles and use hospital quality improvement department to train staff involved in community engagement work.	“We co-locate our food work and our navigation work [at schools] to really make sure that we’re not duplicating efforts, and that we’re reducing the barriers for families, and they’re not confused about where they can go to get some help with these resources” “I do a lot of work in … community relations in terms of collaborating, bringing partners together to help us be more efficient at our work, and helping bridge the gap between the community programming folks and community strategy and grant admin team.” “Every meeting that we have with a partner, we say, “What would you like us to start doing? What would you like us to stop doing? And what would you like for us to continue doing?” And it is through that feedback-rich culture with our partners that we seek to understand how they want us to show up differently.” “We don’t come empty-handed … We say, ‘we understand that it’s going to take some of your staff’s time … How can we support you? What do you need? Do you need a memorandum of understanding? Do we need to pay your staff salary for a week if they help us do this?’” “We are primarily grant and philanthropically funded. I have a team of grant specialists that oversee our different grants from the writing of the grants to the compliance and budgetary constraints as well as program evaluation.” “Another investment the hospital made last year was … to push a bill in [state] that would help reimburse for community health navigators.” “Quality improvement isn’t used to necessarily working in the population health arena and vice versa … And yet we know the critical importance of quality improvement in all that we are doing. So much of our work is focused on PDSA cycles and formal quality improvement methodologies. And we even engage with others to whom we provide technical assistance in community-oriented programming.”
*Maintenance* Leveraging existing ­hospital resources and support structures Internal funding from hospital to ­continue initiatives Prioritize partnerships that are working well Developing infrastructure beyond individual professionals to keep projects moving forward despite staff turnover. Be flexible with collaboration’s trajectory: look for new opportunities to expand the partnership and be flexible to pivot if resources or priorities shift.	“As soon as we established that partnership, we were able to refer some of those injury questions over to [hospital staff member]. [She] was able to come into some of the schools and do some similar programming and talked about building structure.” “I also find that it’s really key to have philanthropy and government relations involved, because they have the relationships with the legislators, … the funding sources.” “Once we have identified who the partner is … we go through that contracting period and set out our expectations. And then once that’s formalized, that goes into an annual legal review.” “There was no infrastructure in place to support them, to enable them to achieve scaling, to enable them to achieve impact, and enable them to achieve sustainability. So, we wanted to create an … infrastructure that could do that.” “[After the grant funding ends], those deliverables end. The relationships don’t. And so, what what’ll happen is you’ll keep trying to find some way to work together. For example, … we created a philanthropy fund to help support keeping that community health worker.”

**Figure 2:**
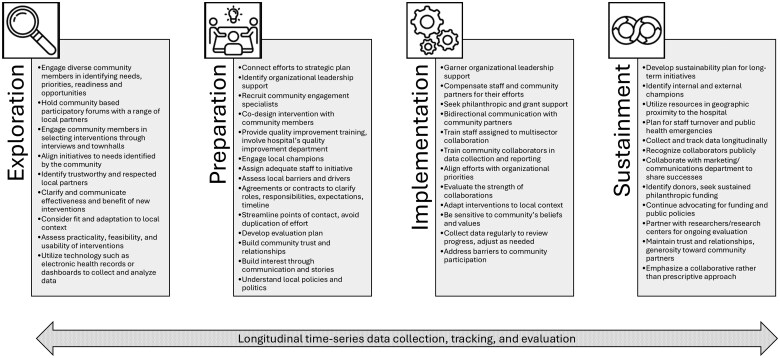
Best practices in implementing multisector collaborations at children’s hospitals

Measuring and tracking the impact of multisector collaborations was reported as key to advancing efforts. Quality measures that hospitals used included reduced delays in accessing health services, decrease in emergency department utilization, increased utilization of prevention services, and access to crisis services. Qualitative data on the effectiveness of multisector collaborations were challenging to obtain; however, one hospital employed a social network analysis tool to measure levels and strengths of collaborations. Other hospitals evaluated their efforts in less formal ways, such as assessing how well initiatives met predefined goals and served families, or considering what aspects of a program should continue, change, or stop. Hospitals reported greater success when they and their community partners agreed about which entity would collect and report data and in what format. In particular, hospitals reported that they benefited from a centralized data collection infrastructure that allowed for transparency and efficiency in evaluating program and health outcomes. Figure 2 summarizes best practices for strategies to implement multisector collaborations.

## Discussion

### Statement of principal findings

Most children’s hospitals in the USA in this study engaged in multisector collaborations aligned with needs identified in their CHNAs, although there is variation in the extent and impact of these activities. Key priorities of hospitals include mental and behavioral health, access to health services, neighborhood safety, violence prevention, early childhood education, and chronic disease prevention. Factors that promote multisector collaborations include adequate staffing and funding, community trust, existing strong partnerships to build on, community engagement specialists, quality improvement training for staff and community collaborators, design sessions to co-create initiatives with community members, and engaging community collaborators in reporting and tracking outcomes data.

Challenges faced include inadequate funding for multisector collaboratives, shortage of staff with training and experience in multisector work, and variation in readiness of community collaborators. Best practices include supporting existing local initiatives, leveraging existing hospital resources, incorporating community partners and financially compensating them for their efforts, building trust and relationships within the community, measuring and tracking the impact of multisector collaborations, and developing the infrastructure to maintain and sustain improvements.

### Interpretation within the context of the wider literature

Although there have been remarkable improvements in pediatric health care quality in the USA, there are still significant gaps in health care delivery and health outcomes. The COVID-19 pandemic exacerbated many of these disparities and increased the urgency of identifying and implementing effective and sustainable solutions [11]. This study identified strategies that children’s hospitals implemented to mitigate health disparities and enhance population health.

Burns *et al*. utilized qualitative content analysis to evaluate the frequency and degree of inclusion of community partners in developing CHNAs from a nationally representative sample of 503 nonprofit hospitals that provide care to adults. Approximately 90% of these hospitals engaged a range of community members in developing CHNA through identifying needs and generating ideas, but only 14% engaged community members in developing or providing feedback on implementation strategies. The authors concluded that hospitals may need additional support and resources to engage communities in selecting interventions [12]. Powell *et al.* [13] also used content analysis to analyze CHNAs of nonprofit general hospitals in Philadelphia and found that assessments most commonly identified health needs related to access to care, however implementation strategies frequently did not address these needs. This study involved content analyses of CHNAs and implementation strategies of children’s hospitals, all of which engaged community members in developing the CHNA and 65% of which integrated community member feedback in developing implementation strategies. Moreover, needs identified in the CHNA aligned well with implementation strategies at 95% of children’s hospitals in the sample. This finding is consistent with financial data that demonstrate that children’s hospitals contribute more resources to community benefit efforts compared to general hospitals. For example, tax-exempt children’s hospitals in the USA spent 17% of their total annual expense on benefits to the community compared to 11% by general hospitals [14]. This high level of community engagement and alignment of implementation strategies with identified needs is a best practice that is conducive to the success or sustainability of interventions to increase multisector collaborations at children’s hospitals.

Children’s hospitals’ CHNAs and implementation strategies in this study included community health initiatives in timely and important areas that impacted care beyond the hospital walls. Traditional quality improvement approaches at children’s hospitals tend to benefit children with access to care which could exacerbate health disparities. Including community health approaches and prioritizing areas identified by CHNAs extends quality improvement efforts to all children and can improve population-level child health outcomes. Published case studies of successful partnerships between children’s hospitals and multisector collaborators demonstrate the range and variation of approaches used by children’s hospitals to increase their impact on child health beyond their hospital walls. For example, the Pediatric Vital Signs Project at Nationwide Children’s Hospital and Franklin County, Ohio enhanced hospital-community partnerships to implement interventions to reduce infant mortality and improve school readiness and high school graduation rates [15]. This study identified a range of successful implementation efforts by children’s hospitals. One hospital utilized the RE-AIM framework to select metrics to track outcomes and to evaluate their improvement strategies. Another hospital created a community engagement guidebook to help hospital staff and community partners design, implement, and evaluate interventions.

### Strengths and limitations

This study highlighted multisector collaborations that can increase the impact of children’s hospitals and extend their reach into the communities they serve. It also identified several feasible and effective initiatives that children’s hospitals can engage in with local schools, social service and housing agencies, faith-based organizations, and public health departments. The study included an analysis of CHNAs that were published by children’s hospitals. In-depth information about specific interventions, implementation approaches, and detailed quantitative outcomes was limited in some cases. Mechanisms that played a role in the success or failure of interventions were sometimes challenging to identify. This challenge was mitigated by expanding on implementation approaches in the in-depth interviews and therefore combined findings from both approaches in the organization and description of these results.

Further in-depth exploration of specific interventions described by hospitals and using quantitative data to assess their outcomes could have been beneficial in evaluating their effectiveness at achieving intended goals. The in-depth interviews probed for context; however, information from a single representative at each hospital in the sample may lead to the potential for personal bias, or misinterpretation or misrepresentation of the respondent’s intent. Respondents shared their hospitals’ barriers and facilitators with the assurance that confidentiality would be maintained during reporting. As a result, this report did not include specific information on local contexts and funders.

### Implications for policy, practice, and research

Next steps include applying the best practices identified to develop a quality improvement learning collaborative to support the implementation of strategies to expand multisector collaborations at children’s hospitals to address child health disparities. Quality improvement learning collaboratives bring together peers from multiple organizations with the goal of improvement in a focused topic area and enable knowledge sharing, implementing evidence-based practices, and interorganizational learning [16]. By focusing on child health beyond the hospital walls, the goal for the learning collaborative will be to identify local gaps in child health, collect data on and track meaningful measures of child health, implement interventions using evidence-based implementation science approaches, and share innovative approaches and learnings, thereby enhancing local improvement capacity. Analyzing aggregate data at the learning collaborative level could help determine which multisector collaborations are most effective and in what contexts, as well as their feasibility and cost-effectiveness.

## Conclusions

Efforts to transform child health care from sick-care systems to community-integrated systems require children’s hospitals to effectively partner with community-based organizations to extend their reach and impact. The results of this study highlight the range of multisector collaborations that children’s hospitals in the USA are engaged in. They additionally identify facilitators and challenges to multisector collaborations, and best practices to implement and sustain such collaborations. The findings of this study can be used to inform the development of strategies to implement future multisector collaborations at children’s hospitals and effectively leverage their strengths and resources to enhance child health and wellbeing.

## Supplementary Material

mzaf067_Supplementary_Data

## Data Availability

Raw data that support the findings of this study are available from the corresponding author, upon reasonable request.
