# Supplementary material for: Multisector collaborations at children’s hospitals to address social drivers of health
Source: Int J Qual Health Care. 2025 Jul 18;37(3):mzaf067. doi: 10.1093/intqhc/mzaf067 (PMC12343070; doi:10.1093/intqhc/mzaf067)
Supplement: mzaf067_Supplementary_Data [file mzaf067_supplementary_data.zip › Table 5.docx]

**Table 5: Examples of multisector collaborations at children’s hospitals**

| **Local school systems**:   - After school activities and mentorship programs - Field trips to community gardens to learn about healthy food - Educational programming around health promotion and suicide prevention - Mental health outreach to schools   **Law enforcement**:   - Child protection team comprising of clinicians, local law enforcement, and justice system   **Social services**:   - Educational classes for parents - On-site presence of community-based organizations for assistance with food security, public assistance, medico-legal partnership, housing, connection with community health navigators. - Partnership with county Special Supplemental Nutrition Program for Women, Infants, and Children (WIC) program - Partnership with the department of child safety to build a foster care clinic   **Advocacy groups**:   - Partnership with health advocacy group for increased healthcare funding and Medicaid expansion - Partnerships with National Association for the Advancement of Colored People (NAACP), Coalition for the Homeless, legal aid organizations, breastfeeding promotion organizations, advocacy group for native/indigenous peoples - Medical-legal partnership where legal aid works with clinicians to increase access to public benefits, housing, and protection for families facing domestic violence. - Partnership with schools, shelters, and advocacy groups working on outreach to unhoused youth.   **Housing**:   - Partnership with local organization that provides public housing and operates a domestic violence shelter - Partnership with an organization that renovates abandoned homes and places vulnerable patients in new housing - Collaboration with a community-based organizations to conduct home visits for children who have visited the Emergency Department for asthma, with the goal of understanding and alleviating triggers in the home   **Transportation**:   - Mobile health unit offers primary and preventive healthcare, such as immunizations, health screenings, and nutrition education - Partnerships with transportation companies (such as local businesses or ride-share mobile applications) to help patients obtain transportation to attend appointments   **Food security**:   - Collaboration with the United States Department of Agriculture to provide free lunches to schoolchildren - Partnership with local farmers markets, supermarkets, and food banks   **Faith based organizations**:   - Partner with local church on emergency food distribution program - Utilize places of worship to disseminate public health information, such as about safe infant sleep practices   **Local or county health and public health departments**:   - Collaboration with local public health department to collect data on local health trends that inform the Community Health Needs Assessment - Partnership with local health department to provide car seats to patients at the children’s hospital - Physician referrals through the electronic health record for home inspection services conducted by the local health department |
| --- |
